# Supplementary material for: Are carbon emissions trading and green financial instruments synergistic? -Comprehensive quantitative research based on content analysis
Source: PLoS One. 2024 Mar 7;19(3):e0298601. doi: 10.1371/journal.pone.0298601 (PMC10919601; doi:10.1371/journal.pone.0298601)
Supplement: S1 Data — (ZIP) [file pone.0298601.s001.zip › Data/地级市Z_F工作报告.zip!/║┌┴·╜¡/┤≤╟∞╩╨/└┤╫╘╣1⁄2╓┌║┼í╛┬φ┐╦╩2╛▌═°í┐_2009.docx]

各位代表：

　　现在，我代表市人民政府，向大会作工作报告，请予审议，并请市政协委员和列席会议的同志提出意见。

　　一、2008年工作回顾

　　过去的一年，我们紧紧围绕“创建百年油田，壮大接续产业，建设宜居城市，构建和谐大庆，全力推进大庆科学和谐跨越发展”的思路目标，加快发展经济、着力改善民生、促进和谐稳定，全市经济社会健康发展，圆满完成了市八届人大三次会议确定的各项任务。

　　——突出好中求快，经济发展迈上新台阶。按照“全党抓发展、中心抓经济、重点抓项目”要求，深入实施“三百”行动计划，培育主导产业，壮大园区规模，狠抓项目建设，经济保持快速发展。全市实现地区生产总值2220亿元，增长12.3%，是近20年发展最快的一年。完成固定资产投资601.9亿元，增长25%；全市地方财政总收入132亿元，增长12.6%；万元GDP综合能耗下降4.8%。工业经济快速增长，规模实力不断壮大。实现增加值1839亿元，增长11.8%。服务油田稳产，生产原油4020万吨、天然气27亿立方米，石油工业实现增加值1448亿元，增长7.8%。支持石化大项目建设，120万吨乙烯改扩建、30万吨聚丙烯等10个重大项目顺利推进，规模以上石化工业实现增加值160亿元，增长16.5%。加速壮大园区化地方工业，开工建设重点项目152个；高新区保持较快发展，完成工业增加值105亿元、增长35.1%，经开区开工千万元以上项目14个，各县区专业园区不断壮大。地方工业实现增加值225亿元，增长35%。现代农业加快发展，粮食生产实现特大丰收。新建抗旱保收田103万亩、玉米膜下滴灌23.2万亩、农机合作社82个，粮食总产达到67.3亿斤，创历史最高水平；奶牛、肉牛、生猪存栏分别增长18.5%、24.5%、24.5%，畜牧业实现增加值35.1亿元、增长9.4%；新型农合组织达到206个，土地规模经营面积近30万亩，农村经济活力不断增强。农业实现增加值69亿元，增长9%。消费需求继续扩大，服务业提档升级。商贸业繁荣发展，大商集团、沃尔玛等大型商场销售额不断攀升，新建大型购物商场40万平方米，新增农资店、农家店200家，社会消费品零售总额实现371.5亿元、增长23%；打造旅游精品项目，成功举办首届湿地文化节，实现旅游收入14.1亿元；服务外包产业园二期工程进展顺利，入园企业达到67家；金融业实现增加值14.2亿元，金融机构贷款比例有所增加，支持地方经济发展有新突破。服务业实现增加值262亿元、增长16.9%。

　　——统筹城乡发展，新农村建设成效显著。农村生活环境有新改善。改造泥草房157万平方米，1.8万户农民住上新居。新建乡镇客运站4个，建设改造通乡村公路1529公里，农村路网框架基本形成。新建饮水安全工程143处、户用沼气1.1万户，新增有线电视4.7万户。整体绿化村屯235个、乡村道路125条，新建防护林网530个，植树造林9.4万亩。农村社会事业有新进步。改造危旧校舍56所，改扩建乡镇卫生院21所。开展“金色田野”等群众文化活动，四县五区文化信息资源共享中心全部建成。农村社会保障有新拓展。开展农村居民养老保险试点，巩固扩大新型农村合作医疗，参合率达到96.7%。实行农村低保家庭大病救助、大学生救助、取暖补助，低保达到全省最高标准。

　　——加快建设步伐，城市功能形象明显提升。46项城建重点工程顺利推进，完成投资129.5亿元。加快构建立体交通体系。铁路大庆西站、哈大齐客运专线、大广高速、大齐高速大庆段全面启动，萨尔图机场快速建设，经九街全线贯通，城区内第一条高速公路机场路建成通车，让通线K409、K418两座跨线桥投入使用，城区主要铁路道口实现立交。加强公用基础设施建设。陈家大院泡生活污水处理厂开工建设，东城区二期、八百垧、乙烯等3座生活污水处理厂和11座垃圾中转站投入使用，城市生活污水和垃圾无害化处理能力明显提高。着力提升城市管理水平。深入开展市容环境综合整治，扩大数字城管系统覆盖面，城市保洁、供热供水、物业服务和交通秩序有新改善。拆除房屋110万平方米，绿化面积225公顷，46公里城区铁路沿线整治全面完成。大规模改善生态环境。建设“油田百里生态长廊”，完成黎明河综合整治一期工程，实施道路景观绿化，植树190万株，新建城市绿地1100公顷，建成区绿化覆盖率达到40%，人均公共绿地10.7平方米，空气质量优良天数356天，顺利通过国家环保模范城复检。

　　——深入推进改革开放，发展活力明显增强。关键领域改革取得重要进展。深化事权改革，向县区下放经济发展、社会管理等领域事权70项，市、县（区）行政效能和服务水平进一步提升；深化财政管理体制改革，启动国库集中支付制度，建立国库单一账户体系，推行公务卡结算，财政支出方式进一步规范；深化国有企业改革，改制重组国有粮食企业19户，国有资产运行效益进一步提高；深化城建项目管理体制改革，完善出台项目组织实施、工程监理和招投标等管理规则，建筑市场管理力度进一步加大。对外贸易实现平稳增长。积极克服金融危机影响，实现进出口贸易总额8.2亿美元、增长20.1%，实际利用外资1.2亿美元、增长26.3%。晟凯毛毯、力神电泵被评为省级出口名牌产品。招商引资取得明显成效。全年共引进项目489个，到位资金116亿元，连续四年突破百亿元。其中，新引进亿元以上项目38个，10亿元以上项目2个，资金到位率和引进项目质量稳步提升。企业上市步伐不断加快。20户企业进入上市储备库，庆客隆、隆赫达和大正公司3家企业在境外顺利上市，全市上市企业达到13家，累计融资40亿元。上市企业天圜营养集团有限公司日月星品牌成为中国驰名商标。

　　——不断提高公共服务水平，社会事业长足进步。努力办人民满意的教育。新建扩建世纪阳光、奥林等学校4所，改造市区相对薄弱学校40所，继农村后全部免除城市义务教育阶段学杂费，市区实现“双高普九”。大力发展优质高中教育，高考成绩连续6年保持全省领先。继续发展高等教育，万人在校大学生达到244人，超过全国平均水平。大庆职业学院进入国家百所示范性高职院校行列，建设中专通过国家级重点中等职业学校评估。加强公共医疗卫生体系建设。市中医院新院、油田总医院住院三部完成主体工程，心脑血管、眼病治疗等重点专科建设取得明显进展；社区卫生服务机构发展到190个，覆盖率达到96%；妥善筛查救治三聚氰胺问题奶粉患儿，有效控制手足口病疫情，我市被确定为省公共卫生建设示范区。大力推进科技创新。实施市级科技计划项目118个，科技进步对经济增长的贡献率达到47.8%。加快推进产学研联合，成功嫁接项目102个。引进省科学院在大庆建设分院，依托八一农大、省化工研究院，联合中直大企业，建设中加食品研发中心、石化产品深加工研究院和装备制造研究院。全面繁荣文体事业。大庆博物馆建成开放，《丹顶鹤》、《经典永恒》等文化精品深受欢迎，“大庆之冬”、“激情之夏”等群众活动广泛开展。北京奥运会和哈尔滨大冬会圣火在我市成功传递，我市举重运动员曹磊在奥运会上勇夺金牌。

　　——着力改善民生，人民生活水平持续提高。城镇居民人均可支配收入达到16816元、农民人均纯收入达到5549元，分别增长8%和41.6%。统筹推进城乡就业。城镇新增就业3.7万人，新转移农村劳动力2万人，城镇登记失业率控制在4.2%以内。不断提升社会保障水平。启动四县城镇居民基本医疗保险，新参保8.9万人，在全省率先实现城镇居民医疗保险全覆盖；加快“五险”扩面，净增32.6万人次；提高“十位一体”低保综合救助水平，实现城镇低保大病救助与全民医保衔接，发放生活保障金和救助金1.5亿元。着力改善群众生活条件。改造棚户区14处、58万平方米，2930户居民搬进新居；建设经济适用住房23万平方米、解决3100户困难家庭住房问题；完成3.5万户天然气入户，投放液化气和供热补贴4200多万元；发放住房公积金贷款23.4亿元。深入整顿规范市场秩序。开展食品市场专项整治，城市食品安全消费满意度综合评价全省最高；持续开展打击“黑出租”专项行动，查扣违法违规车辆1770台，行业秩序进一步规范；清理整顿建筑市场，整改违规工程44项，建筑市场环境进一步优化。维护社会安全稳定。增加市、县（区）两级领导信访接待日密度，组建市、县（区）两级群众来访调处中心，建立信访稳定督查机制，实行各级领导干部接访包案，进京到省上访量明显下降。加强社会治安综合治理，8类主要刑事案件下降20%，打击刑事犯罪工作全省考评第一。全力保障公共安全，健全完善突发事件应急体系，全年未发生重大安全事故。

　　——努力提高行政效能，政府自身建设不断加强。以“优化工作流程，提高行政效能”活动为载体，努力建设服务型政府。权力运行更加规范，在全省率先完成规范行政处罚自由裁量权工作，细化4643个处罚项目标准。减少办事环节696个，压缩办理时限60%。政务公开更加深入，以行政权力公开为重点，全面推进“阳光政府”建设，扩大教育、卫生等8项公共事业办事公开，开办市民议事厅、绝对现场等媒体栏目，政府各部门与群众互动沟通，限时解决供热、物业、就学、就医等978个热点难点问题。效能监督更加有效，以行政服务中心为平台，构建行政效能电子监察系统，对67个部门和单位560项行政权力和服务事项，进行事前、事中、事后全程在线监督。通过明查暗访，对滥用行政职权、推诿扯皮等10个方面26个问题进行了严肃查处。民主法治更加强化，自觉接受人大法律监督和政协民主监督，认真办理人大代表建议286件、政协委员提案203件。广泛听取社会各界意见，受理群众来信来电13.6万件次，征集人民建议519件。加强农村民主政治建设，完成第八届村委会换届选举，一次选举成功率达到93%。

　　近两年来，我们动员全市力量，集中开展国家卫生城、全国文明城“两城联创”。去年，大庆先后成为全省唯一的国家卫生城市和东北三省地级城市中唯一的全国文明城市。这是全市各方合力共建的结果，是全市人民共同努力的结果。不仅标志着大庆经济社会发展和综合竞争力迈上了新台阶，而且彰显出新时期大庆人的精神风貌和大庆的城市魅力。

　　各位代表，回顾过去一年，我市经济社会发展取得的可喜成就，得益于市委的正确领导，得益于人大、政协和社会各界的监督支持，得益于石油石化企业和驻庆中省直单位的鼎力相助，得益于全市人民的团结奋斗。在此，我代表市政府，向全市人民，向所有为大庆经济社会发展做出贡献的同志们和朋友们，表示崇高的敬意和衷心的感谢！

　　回顾过去一年，我们深刻体会到，做好政府工作，推进科学和谐跨越发展，必须始终不断地解放思想，用先进理念武装干部群众，以干事成事为起点，以创新突破为过程，以人民满意为标准，把思想解放的成果落到实处；必须毫不动摇地扭住发展要务，坚持以项目为核心，按项目抓推进，靠项目论政绩，保证经济社会持续健康发展；必须坚定不移地保障民利改善民生，关注衣食住行，解决重点难点，推进城乡等质，维护公正公平，真正让人民群众得到更多实惠；必须一以贯之地维护发展地企和谐关系，发展共促、责任共担、稳定共抓、城乡共建，合力为大庆谋发展、为百姓建福祉；必须鲜明务实地建设责任政府，培养造就一支对党忠诚、对事业尽责、对人民充满感情的公务员队伍，使政府工作经得起群众和历史的检验。

　　回顾过去一年，我们也清醒地看到，全市经济社会发展还存在一些矛盾和问题，政府工作也有一定差距和不足。主要是，产业结构不够合理，接续产业规模相对弱小，经济转型需要进一步加快；发展方式相对粗放，地方经济的产业层次不高，内生增长机制尚未形成；城市功能不够完善，城市承载能力有待提高；城乡二元结构特征比较明显，农村公共服务滞后，城乡一体化发展任重道远；社会事业发展不够均衡，城乡就业、困难群体救助等民生问题还需要更好解决；经济发展环境仍需改善，政府个别部门和少数工作人员服务意识不强、办事效率不高。对于这些问题，我们一定高度重视，采取切实有效措施加以改进。 二、2009年工作安排

　　2009年，是大庆历史上特殊的一年，油田发现50周年、建市30周年，我们将继往开来、再创新业；2009年，国际国内经济形势尤为复杂，全市发展任务特别繁重，我们要振奋精神、攻坚克难。

　　新的一年，我们面临的考验异常严峻，必须主动进取奋发有为，坚定不移保速度。国际金融危机由虚拟经济向实体经济蔓延，对大庆经济产生一定影响；石油价格大幅波动，近一时期低位运行，经济增长不确定因素明显增多；部分产品出口萎缩，市场需求不旺，波及我市相关产业；消费预期发生变化，暂缓投资、持币待购现象显现，趋优消费放慢。面对特殊形势的严峻考验，我们必须坚定信心，全力以赴保速度，千方百计快增长，并以此托起全市经济发展大势。

　　新的一年，我们面临的机遇十分宝贵，必须抢抓先机乘势而起，齐心合力求发展。国家拉动内需，各方投资4万亿元，我们加快经济结构调整、完善城乡基础设施、解决重大民生问题的机会难得；中央实行结构性减税和宽松金融政策，我们扶持中小企业加快成长壮大的机会难得；沿海出口加工型企业受到较大冲击，国家鼓励加工贸易向内陆转移，我们引进项目培植企业的机会难得；省里把哈大齐工业走廊列为全省“八大经济区”之首，明确提出保石油、保石化、保装备，我们加快发展接续产业的机会难得。面对千载难逢的发展机遇，我们必须主动抢位赢得先机，多方联动八方争取，乘势而上突破发展。

　　新的一年，我们肩负的责任重大光荣，必须尽心尽力服务大局，全力以赴保稳产。实现大庆油田储采平衡，保持原油4000万吨持续稳产，是中央的明确要求，是中油集团的战略重点，是全省经济的重要支撑，是大庆发展的基石，也是全市人民的福祉。保稳产，事关国家能源安全，事关大庆长久繁荣，是我们的经济责任，也是政治使命。全市上下必须旗帜鲜明力保4000万吨，坚定不移支持4000万吨，竭尽所能服务4000万吨。

　　新的一年，我们肩负的任务尤为艰巨，必须勇于承担负重奋进，励精图治创新业。省委要求大庆必须超常规、跳跃式、大跨步发展，起领跑作用，走在全省乃至全国前列；市委全会确定打好攻坚战，经济增长“保10争12”，加快由经济大市向经济强市、由生态大市向宜居城市、由矿区城市向现代都市、由总体小康向全面小康、由发展不够均衡向全面协调可持续发展跨越步伐。面对繁重任务，我们必须奋力前行；实现发展目标，我们必须合力攻坚。只要我们继续发扬大庆精神和铁人精神，开拓进取，埋头苦干，就一定能够在新的起点上创造新的业绩。

　　2009年，根据市委七届五次全会确定的“保速度、促发展”工作基调，政府工作总的要求是：深入贯彻落实科学发展观，按照市委总体部署，加快发展保速度，抢抓机遇快建设，多办实事重民生，维护稳定促和谐，努力开创大庆科学和谐跨越发展新局面。主要预期指标：地区生产总值确保增长10%、力争达到12%，固定资产投资增长25%，城镇居民人均可支配收入和农民人均纯收入分别增长6%和17%，城镇登记失业率控制在4.5%以内，万元GDP综合能耗下降4%以上。

　　今年政府工作中心任务是：保速度、快建设、重民生、促和谐。保速度，就是要确保原油4000万吨稳产，推进120万吨乙烯改扩建、30万吨聚丙烯等重大石化项目，实现石油石化经济稳步发展。深入实施“三百”行动计划，实现地方工业跨越发展；大力培育新的经济增长点，实现服务业突破发展；建设四大基地，实现现代农业较快发展。快建设，就是要系统规划、整体建设、纵深管理、开放开发，全力推进城建60项重点工程，宜居城市建设迈出坚实步伐。重民生，就是要完善社会保障体系，全面发展科教文卫体各项事业，落实市委提出的十项民生工程，重点抓好利民惠民实事，人民生活再有新改观。促和谐，就是要坚持“四大”工作机制，实现“两保”工作目标，建设“平安大庆”，维护社会和谐稳定。

　　（一）服务油化经济，筑牢大庆发展基石。坚持主动服务、高效服务，全力以赴支持油田企业开展新时期高科技新会战，打好油气勘探、老区挖潜、外围开发、海塔增储“四个攻坚战”，确保原油4000万吨稳产；全力以赴支持石油石化企业建设全国重要的油气生产、石油化工、科技创新、工程服务和石油石化装备制造“五大基地”；全力以赴支持中直石化企业实施120万吨乙烯改扩建、30万吨聚丙烯等大项目建设，实现主营业务稳步增长。创造更好的政治环境。切实增强服务大企业的政治责任感，凡是油田产能建设和石油石化企业发展需要的，都要想方设法予以支持。结合庆祝油田发现50周年，深入宣传4000万吨稳产和建设“五大基地”的重大意义、石油石化大企业的突出贡献和主体作用，形成全社会关心大企业、保护大企业、支持大企业、服务大企业的浓厚氛围。创造优良的生产环境。适应新形势需要，在帮助解决打井占地、规划审批、项目建设等经常性问题上，要快捷高效；在解决企业发展的特殊问题上，要特事特办；在维护生产经营秩序问题上，要打击偷盗原油行为，排查安全生产隐患，始终保持高压态势。创造方便的生活环境。加快完善大企业所在区域城市功能，重点建好铁路大庆西站及站前新区、西城区体育馆、一中灯岗立交桥和青龙山地区基础设施；提供政策扶持和帮助，改善职工居住环境，支持建好银浪新区和石化职工居住新区；完善社区服务功能，提供更高水平的公共服务。

　　（二）采取超常举措，推动地方工业跨越发展。全力推进“三百”行动计划，向主导产业要规模、向产业园区要速度、向骨干企业要增长、向新建项目要增量、向科技创新要后劲，地方工业增加值增长60%。分产业寻求重点突破。围绕调整优化产业结构，集中力量培育主导接续产业。地方石化深加工领域，主要是集中推进聚烯烃、甲基叔丁基醚和醋酸三大产业链，大力发展塑料加工、橡胶制品和涂料油漆产品，在延伸吃配上突破；农牧加工领域，主要是做大基地规模，做强龙头企业，做精市场品牌，在多元加工、规模加工和精深加工上突破；装备制造领域，主要是整合地企资源，开拓外埠市场，壮大经济总量，在提升规模层次上突破；新型建材领域，主要是做好专业园区，培育大型企业，提升技术水平，在节能环保和集群发展上突破。分层次提升工业园区。整体扩张高新区，加大项目引进和建设力度，快上高新技术产业，高标准建设大豆工业园、石油石化装备制造园、金融产业园和生物技术产业园，力争两年内高新区工业增加值实现倍增；迅速壮大经开区，加快建设温州工业园和闽南工业公寓，开工建设华能电厂热电联供项目，搞好后四厂搬迁入园，争取年内新入驻千万元以上企业20家；全面启动庆南新城产业区，加快完善基础设施，创造项目摆放条件；加强县区专业特色园区建设，不断增强产业集聚功能，加快项目引进，保投产、促达产，实现速度快速增长、规模快速扩张。分类别推进重点项目。尽快启动港荣铝业、汉能二甲醚等107个新建项目，争取早日开工；加快推进200万吨玉米深加工、100万吨甜菜加工、4万吨甲乙酮等44个续建项目，确保如期建成投产；抓紧落实航天科技集团高档泵、精密阀等跟踪洽谈项目，力促尽快实施。分领域培育骨干企业。支持中蓝石化、杜尔伯特伊利等优势企业做大做强，鼓励大丰科技、汉维长垣等成长性好的企业开拓市场，争取肇融集团、龙达纺织、瑞好集团等4家企业上市，引导华科、碧港等企业申报中国驰名商标，奥维电缆、三维科技等企业争创知名品牌。分功能强化科技创新。发挥高新区引领作用，加强创业服务中心、软件园等科技孵化器和中试基地建设，完善科技园区、生产力促进中心等载体功能，打造创新平台；发挥高校和科研院所主导作用，建好石油学院、八一农大科技园和省科学院大庆分院，搞好已立项科技项目研发推广；发挥企业主体作用，引导地方企业建立研发中心、技术中心，促进产学研结合，扩大科技成果转化。利用新技术，大力发展风力发电、地热利用等新能源项目，继续抓好节能减排，积极推进清洁生产，努力发展循环经济。

　　（三）发展农村经济，大力推进社会主义新农村建设。全面贯彻十七届三中全会精神，发展现代农业，培育新型农民，建设富庶文明新农村。农业增加值增长10%以上。壮大工业化引领的县域经济。围绕粮资源、畜资源、水资源，做大、做强、做优乳业、薯业、皮革业、麻纺业和松嫩两江水产业，发展大龙头，形成企业群，带动大基地，壮大县域经济规模。积极争取大企业支持，搞好小油田开发，增强县级财政实力。打造基地化的现代农业。建设百亿斤粮食生产基地，加大农业基础设施建设力度，新建玉米膜下滴灌50万亩、喷灌53万亩，新建农机合作社37个，抗旱保收田达到566万亩，高产优质粮食发展到450万亩，粮食产量达到75亿斤。建设安全优质牧业生产基地，以奶牛、生猪、大鹅为重点，标准化养殖小区发展到190个，养殖大户发展到7500个，提高畜禽良种覆盖率，建设完善防疫安全、市场安全和食品安全体系，促进畜牧业扩规模、提质量、增效益。建设无公害绿色食品基地，对同类作物产品，逐步统一生产标准、统一规模经营、统一品牌培育、统一市场开发，壮大产业规模，提高产业化水平，基地面积发展到400万亩。建设区域观光农业基地，推进农业和旅游业组合开发，着力打造东、西干线观光农业长廊，建设沿江、沿湿地生态农业旅游观光带，力争建成全省重要的旅游观光农业区。扩大市场化的经营方式。落实完善扶持政策，大力发展农村合作经济组织，支持引导向更宽领域拓展，向多村多乡联合发展，向公司制、集团化管理方向迈进，推动与龙头企业建立紧密型利益联结机制，农合组织发展到260个以上。鼓励农民以转包、出租、互换、转让、股份合作等形式流转土地承包经营权，扩大土地规模经营。建设规范化的农村基础设施。继续完善县乡村基础设施规划，支持四县县城建设生活垃圾、污水处理厂，加强村镇主次干道、给水排水等配套设施建设，开展村屯环境综合治理。新建乡镇客运站6个、农村公路500公里、农村饮水安全工程252处，新增户用沼气1万户、农村有线电视3.5万户。植树造林12万亩，整体绿化村屯260个。加快农村城镇化步伐，支持有条件的县区开展城乡一体化试点。 （四）着眼城乡繁荣，壮大提升服务业。适应城市转型需要，培育壮大现代服务业，服务业增加值增长30%以上。组合开发商贸业。引进国内外知名开发企业，集中开发建设集特色商住、特色商业、特色餐饮、特色观光于一体的商贸新城，打造都市特色商贸品牌。继续引进名企名店，支持鼓励沃尔玛、大商、庆客隆等大型商贸企业向社区延伸，新增社区连锁超市20家，力争西城大润发购物广场建成开业。整体做活物流业。支持石油物流园、神舟石化物流园开发建设，扩大市场份额，增强拉动力。做好地方物流业发展规划，整合提升物流龙头企业，抓紧辟建区域物流园区。大力发展小商品、建材、农资批发，做大做强新站粮食、中九路粮油等批发市场。加快壮大服务外包业。以高新区服务外包产业园为依托，突出石油工程技术服务、软件开发、信息处理等重点，着力引进大型企业，培育壮大以华拓数码、银象集团为骨干的企业群体，实现产业规模和层次整体提升。大力发展金融业。深化银企对接合作，促进金融和经济深度融合，引导鼓励金融机构灵活创新金融产品，扩大信贷投放；加强社会征信体系建设，完善多元化担保体系，壮大市属国有资本担保平台，鼓励支持社会力量组建商业性、互助性和行业性担保公司；把金融业作为重要接续产业，加快建设金融产业园，开放金融业，做活保险业和证券业，发展壮大金融信托与管理、金融租赁、典当业务。深度开发旅游业。整合石油文化、湿地风光、绿色温泉、草原风情、观光农业等特色旅游资源，组合规划路线，整体包装推介，建设完善景区项目15个，在深度开发资源上突破；高水平办好第二届湿地文化节，与国内外知名旅游城市和旅游中介全面合作，在拓展旅游市场上突破；促进吃住行游购娱关联发展，开展评选十佳餐饮、十佳宾馆、十佳旅游纪念品经销、十佳诚信旅游企业活动，增强旅游地接能力，在提升配套服务上突破。力争年接待游客700万人次以上，实现旅游总收入20亿元以上。积极扩大城乡消费。制定出台刺激消费的政策措施，增强居民消费信心，进一步激活房地产、汽车、旅游等市场，促进城市消费；扩大“万村千乡”便民连锁，加快推进“家电下乡”，拓展农村消费。

（五）创新提升理念，努力加快宜居城市建设。突出宜发展、宜创业、宜生活、宜生态、宜文化的宜居城市理念，拓展城市空间，完善城市功能，提升城市形象，增强人民群众的舒适感、归属感和满意度。系统规划设计城市。修编完善城市总体规划，解决好区域定位、城市特色、空间布局和功能分区等重大问题。紧密衔接总体规划，加快完善道路、给排水、供电、供热、通讯、绿地、地表水系等专项规划，高起点做好重点开发区块控详规划和重点区域城市设计。把今年作为规划编制年，在完成已确定的10项重点规划基础上，进一步加大规划编制力度，年内基本形成完整的规划体系。面向国内外聘名家、请名院，把规划做成精品；实行专家制定、社会参与、多方审查、依法审批，真正体现人民城市人民规划。整体开发建设新区。实施“南进”战略，依托红岗、大同、林源三个现有城区，规划建设庆南新城，今年重点构建新区基本框架，配套完善基础设施，启动建设庆南新城产业区。加快“北扩”进程，依托五湖地区、萨尔图机场，规划建设庆北新城，今年重点建好水电路讯。提升东西两个城区的形象和功能，加大北国之春梦幻城项目推进力度，加快青龙山地区、铁路大庆西站站前地区等重点区块的整体开发。精心建设重点项目。加快实施60项城市建设重点工程。抓好大广高速、大齐高速、萨大路改造等路桥项目。搞好城市轨道交通、铁路大庆东站论证规划，配合建设哈大齐客运专线，加快建设铁路大庆西站、公路客运枢纽工程，确保萨尔图机场和森警直升机场年内通航。启动建设城市规划展示馆、奥林匹克公园等项目。建好东城水库、主城区生活垃圾无害化处理厂，新建10座垃圾中转站。继续搞好东方玫瑰园、奥林国际公寓等居住区开发，力争房地产开发面积达到570万平方米。推进城市纵深管理。坚持建管并重，理顺管理体制，制定管理标准，强化行业管理，落实属地责任，形成运转高效的城管机制。加快城管监控系统和公安监控系统全面整合，实现主城区数字化管理全覆盖。巩固国家卫生城和全国文明城创建成果，集中开展市容市貌、城乡结合部和居住区、交通秩序、公共聚集场所、建筑工地、集贸市场、景点景区、铁路沿线、油田矿区、城市空闲地“十项城市环境综合整治”，形成长效管理机制，提升城市管理水平。规范开放建设市场。引进国内著名房地产开发企业、实力雄厚的建筑企业和经验丰富的监理公司，参与城市建设，整体提升开发建设水平。开展建筑市场整顿，规范开发行为，形成公开公平公正的市场秩序；本着“好、快、省”的原则，加强政府投资项目管理，确保项目建设的质量和标准。加大生态建设力度。全方位、大规模进行生态治理。老区还建生态，找空建绿、见缝插绿、拆废还绿，全面推进城区绿化整理；新区构建生态，综合考虑新区水系、林带、绿网等配套建设，力求环境景观一步到位。推进重点工程，搞好油田百里生态长廊、石化防护林带和环城林网建设，新建城市绿地1300公顷，植树140万株。在完成黎明河水系综合整治的基础上，建成开放黎明河带状公园，改造青龙湖、兰德湖、三永湖、东水源泡和北伊哈齐泡，全力打造区域生态绿化景观带。

　　（六）构筑体制机制，深入推进改革开放。加快重点领域改革。进一步完善事权改革，强化指导服务，逐步建立起便民高效的行政管理体制。深化驻外办事机构改革，在现有职能调整基础上，实行事企分开。推进市属国有企业改革，基本完成市属粮食企业改制，改革投资、担保体制机制，扩大融资功能，健全完善国有资产保值增值考核体系。积极引进各类人才。放宽放活人才引进政策，大力引进各领域急需的高端人才。降低大学毕业生进入门槛，搭建创业平台，引导大学生自主创业、灵活就业。强力推进招商引资。优化招商政策，做好园区载体，整体引进企业，大批引进项目。加强与大型国有企业沟通联系，争取更多的中央企业投资项目落户大庆。与驻庆石油石化大企业共同盘活存量资产，多上关联产业项目。创新招商引资方式，在现有的工业园区内辟建专业园区，探索整体招商；加强与深圳贸促会等国内外中介机构联系合作，降低招商成本，提高招商效率，在中介招商上取得实质性突破。落实招商责任，实行严格奖惩，全年引进亿元以上大项目30个，到位资金100亿元以上。大力发展民营经济。深入实施“中小企业成长工程”，加强分类指导、分层推进、分级包保，不断提升民营经济的规模和质量，使其成为吸纳劳动就业、促进地方经济增长的重要力量。继续优化经济发展环境，强化“改善经济发展环境投诉中心”作用，研究制定鼓励创业优惠政策，逐步形成全民创业的良好氛围。稳步扩大对外贸易。加快出口加工区和内陆港报批建设，优化外贸出口环境。突出俄罗斯、韩国等重点国家和地区，巩固发展农产品深加工、纺织、精细化工、石油机械设备等出口基地。抓住高新区成为“国家科技兴贸创新基地”的有利契机，培育建设高科技产品出口基地，壮大外贸出口规模。支持鼓励晟凯毛毯、力神泵业等企业走出去，开展境外加工贸易，开拓国际市场，扶持发展外贸骨干企业。力争外贸进出口总额增长15%。

　　（七）着力改善民生，努力加快和谐社会建设。在“保速度、促发展”的同时，更要重视民生民利，大力发展社会事业。均衡发展教育事业。规范发展幼儿教育，提升发展义务教育，优化发展高中教育，加快发展职业教育，支持发展高等教育。按照积极稳妥、分步实施的原则，推进教育资源整合，逐步实现全市教育资源整体优化配置。规范幼儿教育管理，优化教育教学内容，提高幼儿教师素质。推进现有义务教育学校标准化建设，建好兰德、青龙山、高新区等九年一贯制学校，力争2—3所高中晋升省级示范高中，市区学生享受优质教育比例达到80%。支持高校稳定招生规模，打造名牌学科、名牌专业，提高办学水平。整合职教资源，扩大职教规模，推进学历教育与技能教育紧密结合，完善实习实训基地，培养产业发展急需的实用技能人才。加强公共医疗卫生建设。改善医疗条件，油田总医院住院三部、市中医院新院投入使用；提升综合医院水平，扩大油田总医院、哈医大五院辐射能力；支持发展重点专科，加快建设眼病、心脑血管等8大医疗特色服务中心；实施社区卫生服务机构“标准化建设工程”，完善县乡村三级医疗网络，综合提升新农合保障能力，适当增加报销病种、提高大病补偿标准。繁荣文化体育事业。以传承弘扬大庆精神、铁人精神为核心，以建国60周年、油田发现50周年和建市30周年为契机，深入开展系列庆祝活动，展示大庆发展成就，提高城市影响力和知名度。辟建文化创意产业园，建成石油科技博物馆，完善大庆博物馆，发挥歌剧院功能，搞好演出市场开发。推进乡镇综合文化站建设，搞好“激情之夏”、“大庆之冬”等群众文化活动，开展全民健身运动，办好第十届市运会。完善社会保障体系。不断扩大城乡社会保障覆盖范围，在做好各险种扩面的基础上，重点抓好城市低保 “十位一体”综合救助提标、农村社会养老保险试点扩面和农村低保救助体系建设，着力解决孤老孤儿、残疾人、重症病人、特困户、五保户等特殊群体的生活问题。加大住房公积金归集扩面力度，扩大贷款发放规模，促进职工改善居住条件。提升社区服务功能。完善社区医疗卫生便民服务，建立居民保健服务体系；完善社区文化娱乐场所和设施，丰富居民文体生活；完善社区治安防控体系，提升居民安全感。努力建设平安大庆。围绕“两保”工作目标，深化落实“大信访、大调解、大防控、大安全”工作机制，重点开展四个专项行动。以化解矛盾、解决积案为重点，开展信访调处专项行动；以保障企业生产安全为重点，开展危险化学品安全治理专项行动；以畅通高效、减少事故为重点，开展交通秩序整治专项行动；以健全打防控一体化体系为重点，开展社会治安治理专项行动，确保社会平安稳定。

　　在全面加强社会建设的同时，努力增加财力物力投入，具体办好与群众生活密切相关的九件事。一是百姓安居，抓好以萨中地区为重点的50万平方米棚户区改造，完成4.14万户农村泥草房改造任务，建设廉租房、经济适用住房30万平方米以上。二是老区还建，结合城区整理，在主城区选择15处闲置公建和裸露地，还建“一库三场”，地下建设公共停车库，地上建设运动场、休闲广场和停车场。三是道路整理，维修、改造、扩建主城区街路30条、63公里，提高城市通行效率。四是燃气入户，完成东西两个城区天然气入户收尾，抓好红岗、大同2.5万户天然气入户工程，力争年内全部完工。五是稳定就业，建立城乡统一的人力资源市场，落实创业就业政策，以创业带就业，开发城镇就业岗位5万个；开展“普及实用技术、培育新型农民”活动，新转移农村劳动力2万人。六是完善社会养老体系，适应人口老龄化趋势，完善市第一、第二福利院，整体提升29个农村敬老院，搞好公办养老；私立老年公寓发展到30家，扩大社会养老；抓好10个社区居家养老综合配套服务试点，普及居家养老。七是建设人才公寓，在完善外来人口居住区的同时，重点建设一批小户型的人才公寓，通过梯次收费、定期迁出、循环周转的办法，解决大学毕业生临时居住问题，提高城市人才吸引力。八是实施乡院村医提升工程，培训村医500名，3年完成全部村医培训；改造提升10所以上乡镇卫生院，力争3年乡镇卫生院全部达到国家标准。九是开展食品药品安全集中整治，坚决打击制假、售假行为，坚决查处各类质量不达标产品，坚决取缔不合格的小作坊、小工厂，切实保障城乡群众利益。

　　（八）加强政府建设，增强推动科学和谐跨越发展的能力。面对复杂多变的经济形势，面对保速度、促发展的艰巨任务，面对全市人民的期盼重托，各级政府必须保持清醒头脑，坚定必胜信心。要以开展深入学习实践科学发展观活动为动力，切实加强政府自身建设，努力做到行政提速、管理创新、执行彻底、结果问效，全面完成今年各项任务目标。树立雷厉风行的工作作风，确保行政提速。大力倡导“马上行动”的工作理念，切实转变工作作风，坚决消除中梗阻、推拖靠、走过场等现象，该干的事快定、定完的事快办，以只争朝夕的精神和时间赛跑，始终保持工作高效率、快节奏。大力倡导求真务实之风，改进文风、会风，压缩、精简会议和文件，集中精力谋事干事抓落实。建立高效透明的运转机制，实现管理创新。改进工作机制，开展“政务创新”活动。推进思维创新，把思维的起点放在成事上，把思维的重点放在突破难点上，把思维的着力点放在敢想敢干上；推进流程创新，继续精简行政审批，大胆探索并联审批，缩短办事环节，提高行政效率；推进方式创新，扩大电子政务，在更多领域加快信息化建设，增强政府网上办事功能。落实国家取消和暂停100项行政事业收费政策，认真开展收费项目清理，大力减轻企业和群众负担。形成畅通协调的工作格局，做到执行彻底。全面增强各级政府公信力、执行力，说到就要做到，做了就要做好，努力让企业基层满意、让人民群众满意。保持政令畅通，把有令则行、有禁必止作为政治纪律，彻底消除工作运行中的梗阻、掣肘，确保决策和政令落实到底。狠抓奖惩分明的绩效监察，严格结果问效。继续抓好“优化工作流程、提高行政效能”活动，强化效能监察，量化效能评价，建立优劣分明的效能评价机制。加强廉政建设和公务员队伍建设，大张旗鼓地树立宣传恪尽职守、热忱服务、甘于奉献的先进典型，严肃处理吃拿卡要、敷衍塞责、以权谋私的反面典型，提高各级政府和公务人员为基层服务、为人民服务的能力和水平。

　　始终坚持市委统一领导，主动接受人大法律监督，自觉接受政协民主监督。认真贯彻落实市委决策，认真执行市人大及其常委会重要决议，认真办理人大代表建议和政协委员提案，支持工会、共青团、妇联等群团组织开展工作。

　　各位代表，做好政府工作，推动大庆经济社会更好更快发展，我们肩负的使命重要而光荣。我们将始终坚持“负责任地干事、干负责任的事”，在市委的正确领导下，弘扬大庆精神、铁人精神，团结依靠全市各族人民，解放思想，奋发进取，迎难而上，扎实工作，为实现大庆科学和谐跨越发展而努力奋斗！
